# PCR-hybridization after sonication improves diagnosis of implant-related infection

**DOI:** 10.3109/17453674.2012.693019

**Published:** 2012-06-04

**Authors:** Jaime Esteban, Noelia Alonso-Rodriguez, Gema del-Prado, Alberto Ortiz-Pérez, Diana Molina-Manso, Jose Cordero-Ampuero, Enrique Sandoval, Ricardo Fernández-Roblas, Enrique Gómez-Barrena

**Affiliations:** ^1^Department of Clinical Microbiology; ^2^Bone and Joint Infection Unit, IIS-Fundacion Jimenez Diaz; ^3^Department of Orthopedics, Hospital Universitario La Princesa; ^4^Department of Orthopedics, IIS-Fundacion Jimenez Diaz; ^5^Department of Orthopedics, IdiPaz-Hospital La Paz, Madrid, Spain; Correspondence JE: jestebanmoreno@gmail.com

## Abstract

**Purpose:**

We wanted to improve the diagnosis of implant-related infection using molecular biological techniques after sonication.

**Methods:**

We studied 258 retrieved implant components (185 prosthetic implants and 73 osteosynthesis implants) from 126 patients. 47 patients had a clinical diagnosis of infection (108 components) and 79 patients did not (150 components). The fluids from sonication of retrieved implants were tested in culture and were also analyzed using a modified commercial PCR kit for detection of Gram-positive and Gram-negative bacteria (GenoType BC; Hain Lifescience) after extraction of the DNA.

**Results:**

38 of 47 patients with a clinical diagnosis of infection were also diagnosed as being infected using culture and/or PCR (35 by culture alone). Also, 24 patients of the 79 cases with no clinical diagnosis of infection were identified microbiologically as being infected (4 by culture, 16 by PCR, and 4 by both culture and PCR). Comparing culture and PCR, positive culture results were obtained in 28 of the 79 patients and positive PCR results were obtained in 35. There were 21 discordant results in patients who were originally clinically diagnosed as being infected and 28 discordant results in patients who had no clinical diagnosis of infection.

**Interpretation:**

For prosthetic joint infections and relative to culture, molecular detection can increase (by one tenth) the number of patients diagnosed as having an infection. Positive results from patients who have no clinical diagnosis of infection must be interpreted carefully.

Management of orthopedic implant-related infections starts with a proper etiological diagnosis, which is required for specific antibiotic treatment. Different approaches are used to obtain such a diagnosis (Trampuz et al. 2006, Del Pozo and Patel 2009) and these must take into account the importance of the development of bacterial biofilms in the pathogenesis and management of implant-related infections (Trampuz et al. 2003, 2006, Costerton 2005).

The use of low-intensity ultrasound that releases biofilms is an alternative to classical culture methods from implants, and several protocols have been developed for this purpose (Trampuz et al. 2007, Dora et al. 2008, Esteban et al. 2008, Piper et al. 2009, Achermann et al. 2010). In these reports, the use of sonication of retrieved implants was reported to have similar sensitivity to or higher sensitivity than conventional techniques. Nevertheless, there are still patients with a clinical diagnosis of infection and negative cultures (Berbari et al. 2007). Previous use of antibiotics has been implicated as one of the main causes of this problem (Trampuz et al. 2007), but other causes are also possible. To solve the problem, molecular biological techniques have been proposed in order to obtain faster and more accurate results than conventional culture (Tunney et al. 1999, Sauer et al. 2005, Dempsey et al. 2007, Fihman et al. 2007, Moojen et al. 2007, Gallo et al. 2008, Kobayashi et al. 2008, Vandercam et al. 2008, De Man et al. 2009, Piper et al. 2009, Achermann et al. 2010, Riggio et al. 2010, Marin et al. 2012). Most of these reports were based on protocols that were developed in-house, which are difficult to integrate into clinical microbiology routines, even though they may give good results. Recently, however, commercial kits have been designed to work under common routine laboratory conditions. Here, we describe a study on the diagnosis of infection in a broad range of orthopedic implant-related infections, comparing conventional culture with detection of microbial DNA using a commercial kit—in both cases after sonication of retrieved implants.

## Methods

### Patients

We included 126 consecutive patients undergoing surgery with retrieved major orthopedic implants (185 prosthesis implants and 73 osteosynthesis implants) between 2004 and 2009. These patients were treated in 2 university hospitals in the Madrid area. Clinical diagnosis of implant-related infection was performed according to 1 of 2 internationally accepted criteria: (1) draining sinus, or (2) inexplicable pain, persistent local erythema plus swelling, CRP greater than 1 mg/dL, and ESR greater than 30 mm in the first hour (Cordero-Ampuero et al. 2007). Data on antibiotic treatment since device implantation surgery or any other treatment during the month before the surgery were also recorded for each patient.

The ethics committee of Fundacion Jimenez Diaz approved the study (March 30, 2010; registration number PIC 05/2010).

### Implant types

We processed 258 orthopedic implants (2 implants per patient), including 84 hip and 101 knee prosthesis components (from 40 and 35 patients, respectively), 41 intramedullary nails and screws (from 29 patients), and 32 other osteosynthesis implants (multiple screws, dynamic screws, plates; from 22 patients).

### Implant processing

Implants were aseptically removed from the patient, in all cases during revision surgery. These were placed in 3 sterile bags in the operating room and sent to the microbiology laboratory within 24 h. There, each of the components was processed according to a previously described method (Esteban et al. 2008), which had a detection limit of 100 CFU/mL. Clean distilled water was added to the sonicator before processing the sample, and then discharged after sonication to minimize the risk of contamination. Aliquots of the resuspended sonicate (2 vials of 1 mL) were frozen at –80ºC to await DNA extraction.

Identification of isolates was done using conventional methods (API strips; bioMérieux, Marcy l’Etoile, France). Susceptibility testing was performed using internationally recommended techniques for each species (Clinical_Laboratory_Standards_Institute_(CLSI) 2009). The isolation of 3 or more different anaerobic species was recorded as mixed anaerobic microbiota, without any further attempts at identification.

Environmental bacteria that are uncommonly isolated from human infections and which appeared in low counts in the cultures were considered to be potential contaminants due to the technique. Leaking bags were also considered to be contaminated. Random cultures were performed from the distilled water used in the sonicator to identify potential sources of contamination, but no positive results were obtained from these cultures.

### DNA extraction and PCR studies

DNA extraction was performed in 2 ways, depending on whether or not blood was present in the fluid obtained after sonication. For samples without any evidence of blood, a method based in immunomagnetic extraction (EasyMag; bioMérieux, Marcy l’Etoile, France) was used, and for those samples containing traces of blood, the commercial kit MolYsis Complete 5 was used (Molzym GmbH & Co. KG, Bremen, Germany). DNA extracts were conserved in a final volume of 50 µL at –20ºC.

PCR studies were performed using the GenoType commercial system BC Grampositive and BC Gramnegative (Hain Lifescience GmbH, Nehren, Germany). This system allows PCR-based amplification of a fragment from the 16 rRNA gene present in the sample and subsequently allows hybridization on a nitrocellulose strip that has specific probes. The combinations of hybridized probes lead to identification of the isolate, and allow detection of the presence of more than 1 species in the sample. We used the kit according to the instructions provided by the supplier, with the following modification. DNA samples were amplified using the PCR protocol as follows: 95ºC for 8 min; 32 cycles of 95ºC for 30 sec, 55ºC for 40 sec, and 72ºC for 40 sec; and a final DNA-elongation phase of 72ºC for 8 min. The PCR products were analyzed by solid-phase hybridization according to the instructions provided by the manufacturer (Eigner et al. 2005).

### Data analysis

To evaluate the accuracy of the molecular detection compared to conventional culture, we considered only concordance with those samples with positive cultures for any of the organisms specified in the kit. To evaluate the usefulness in the diagnosis of infection, we considered positive results of both techniques separately and also the results of both techniques together. Also, we evaluated prosthetic joint infections and osteosynthesis device-related infections separately. Colony counts of ≥ 10^5^ CFU/mL were combined for calculation purposes. Statistical evaluations were performed using EPI-INFO software version 3.5.1 (Centers for Disease Control, Atlanta, GA).

## Results

The mean age of the patients was 66 (23–96) years; two-thirds were women. As expected, the mean age was lower for patients with osteosyntheses than for patients with prostheses (56 vs. 73 years, p < 0.001, Kruskal-Wallis test). All cases of infection were late (i.e. infections that appeared 3 or more weeks after surgery) except 2 cases of *S. aureus,* which were acute prosthetic joint infections.

According to clinical criteria, 47 patients were diagnosed as being infected. A positive history of antibiotic therapy was registered in 32 of them. In patients without any clinical diagnosis of infection, 15 had been treated with antibiotics previously because of other infectious conditions. The patients were divided into 2 groups because of the differences in etiology and pathogenesis of infections in the 2 groups: those with joint prostheses and those with osteosynthesis implants. Potential contaminations were excluded in both groups.

### Microbiological diagnosis

258 samples of sonication fluid (2 samples per patient) were analyzed, 109 of which belonged to patients with a clinical diagnosis of infection. Of these samples, 93 were found to be culture-positive.

*Staphylococcus aureus* and *Staphylococcus epidermidis* were the most commonly identified organisms, being found in 21 and 29 samples, respectively (Tables 1 and 2). For *S. aureus, *20 samples belonged to clinically infected patients and only 1 to a patient who had not been clinically diagnosed as being infected. On the other hand, 3 of the 29 positive samples for *S. epidermidis* corresponded to 3 patients who had not been diagnosed clinically as being infected, and the other 26 samples came from clinically diagnosed infections. Uncommon gram-negative non-fermenting bacilli that may have been potential contaminants were identified in only 13 samples (5% of all the samples).

**Table 1.** Bacterial and fungal species isolated by conventional culture

	Orthopedic implant (no. of samples)				
Bacteria	HPC	KPC	NS	OS	Total
*S. aureus*	9	4	4	4	21
*S. epidermidis*	20	5	4	–	29
*S. warneri*	2	–	2	–	4
*S. hominis*	3	–	–	–	3
*S. lugdunensis*	4	–	–	1	5
*S. milleri*	–	–	2	–	2
*S. pyogenes*	1	–	–	–	1
*K. pneumoniae*	–	4	–	–	4
*M. abscessus*	1	–	–	–	1
*M. fortuitum*	–	–	1 **^a^**	1 **^a^**	2 **^a^**
*Corynebacterium sp.*	–	–	–	1	1
*E. coli*	–	2	–	1	3
*Enterococcus sp.*	–	–	–	1	1
*E. aerogenes*	4	–	–	–	4
*E. faecalis*	–	–	1	1	2
*B. fragilis*	2	–	–	–	2
*P. stuartii*	–	–	1	–	1
*P. prevotii*	–	–	1	–	1
Other anaerobes	–	–	4	1	5
*Micrococcus sp*.	–	–	1 **^a^**	–	1 **^a^**
*P. acnes*	1 **^a^**	–	2	1	4 (1 **^a^**)
*P. aeruginosa*	2 (1 **^a^**)	4	–	–	6 (1 **^a^**)
*P. stutzeri*	–	–	1 **^a^**	–	1 **^a^**
*R. picketti*	–	5 (2 **^a^**)	–	–	5 (2 **^a^**)
*Bacillus sp.*	–	–	1	–	1
*Burkholderia sp.*	4 **^a^**	–	–	1 **^a^**	5 a
*S. maltophilia*	1	–	–	1 **^a^**	2 (1 **^a^**)
*S. paucimobilis*	–	–	1 **^a^**	–	1 **^a^**
*Pasteurella sp.*	–	1 **^a^**	–	–	1 **^a^**
*Prevotella sp.*	1	–	–	–	1
Fungi					
*Candida sp.*	–	–	1	–	1
*A. terreus*	2	–	–	–	2

**^a^** Contaminant microorganisms (of no clinical relevance, low counts). HPC: hip prosthesis components; KPC: knee prosthesis components; NS: nails and screws; OS: other osteosynthesis material.

**Table 2.** Species detected by PCR-hybridization analysis

	Orthopedic implant **^a^** (no. of samples)				
Bacteria	HPC	KPC	NS	OS	Total
*S. aureus*	8	15	5	6	34
*S. epidermidis*	23	17	1	1	42
*S. warneri*	–	1	–	–	1
*S. hominis*	–	3	–	–	3
*S. pyogenes*	2	–	–	–	2
*Streptococcus sp.*	–	–	2	–	2
*H. influenzae*	–	–	2	–	2
*K. pneumoniae*	–	4	–	–	4
*E. coli*	–	3	–	2	5
*E. cloacae / E. aerogenes*	5	1	–	2	8
*E. faecalis*	–	–	–	1	1
*E. casseliflavus*	–	–	–	1	1
*P. aeruginosa*	2	5	–	–	7
*P. mirabilis*	2	–	–	–	2

**^a^** See Table 1

Staphylococcal species were isolated from all groups; *S. aureus* was the only microorganism isolated from all types of implants. Polymicrobial infections were identified from 24 of the 109 samples from infected patients.

The mean colony count was 44 × 10^3^ CFU/mL (range: 50 to > 10^5^). No differences were seen in counts obtained from patients who were diagnosed clinically as being infected (mean: 44 × 10^3^ CFU/mL) and patients who were not (mean: 46 × 10^3^ CFU/mL). No differences were seen between patients with and without previous antibiotic intake (mean counts: 49 × 10^3^ CFU/mL and 36 × 10^3^ CFU/mL, respectively, p = 0.4, ANOVA).

PCR analysis detected 78 positive samples from patients who had been diagnosed clinically as infected and 27 from those who had not (Table 3). Bacteria that were isolated from our samples but which could not be identified with the commercial PCR kit included *S. lugdunensis, B. cepacea, Burkholderia sp., R. picketti, M. fortuitum, M. abscessus, Bacillus sp., S. paucimobilis*, and all the anaerobes.

**Table 3.** Comparison of results from PCR and culture

	Total no. of samples	PCR		Culture	
		+	–	+	–
Clinical infection					
Yes	109	78	31	67	42
No	149	27	122	10	139
Culture					
Positive	77	59	18		
Negative	181	46	135		

### Joint prostheses

To evaluate the usefulness of PCR in diagnosis, we combined the results of both techniques. 28 of the 31 infected patients were diagnosed by taking account of both techniques, giving an increase in positivity of almost one tenth compared to patients diagnosed by conventional culture alone. A higher number of positive samples were detected with PCR than with culture (Tables 4 and 5). Considering patients who were originally categorized as uninfected, the combination of both laboratory techniques gave positive results in 17 cases, an increase of one fourth compared to those diagnosed only by culture. Interestingly, of the patients who were originally classified as uninfected, but who then had a PCR-positive and culture-negative result, 7 cases were positive for *S. aureus.*

**Table 4.** Results obtained from arthroplasty and fracture patients in this series

	Total no. of cases	Culture		PCR	
Patients with retrieved implants		+	–	+	–
All arthroplasty patients under study	75	29	46	40	35
All fracture (nailing and/or osteosynthesis)					
patients under study	51	16	35	16	35
All patients	126	45	81	56	70

**Table 5.** Results obtained from patients with or without clinical diagnosis of infection in our series

	Total no. of cases	Culture		PCR	
Patients with retrieved implants		+	–	+	–
Arthroplasty cases with clinical infection	31	24	7	26	5
Arthroplasty cases without clinical infection	44	5	39	14	30
Fracture cases with clinical infection	16	12	4	8	8
Fracture cases without clinical infection	35	4	31	8	27
Patients with clinical diagnosis of infection	47	36	11	34	13
Patients without clinical diagnosis of infection	79	9	70	22	57

When we analyzed the effects of previous antibiotic therapy, 14 of 20 clinically infected patients showed positive culture results, as compared to 16 of 20 who showed positive PCR results. All culture-positive cases were PCR-positive, and 2 more cases were positive by PCR alone. Of the patients who were not originally diagnosed as being infected, only 1 case was culture-positive and 2 showed a positive result by PCR (1 of them being a culture-positive case).

### Osteosynthesis implants

Combination of the results of both techniques showed positive results in 13 patients who had been clinically diagnosed as being infected (all PCR-positive cases were culture positive) and in 14 patients who had not (2 cases were both PCR-positive and culture-positive), an increase of one fifth regarding positive results in this group of patients.

22 patients had received antibiotic therapy before surgery (12 of whom had already been diagnosed clinically as being infected). Of the 12 cases who had been diagnosed clinically, 9 were culture-positive and 7 were PCR-positive. Of the 10 patients who had not been diagnosed clinically as having an infection, 2 were culture-positive and 5 were PCR-positive.

## Discussion

Most reports on the use of molecular biological tools to detect bacteria in periprosthetic tissues have been based on amplification of a broad-range bacterial target, such as 16S rRNA sequences, and subsequent identification of the organisms detected by sequencing of the fragment. More recently, the use of specific primers to detect specific pathogens has also been evaluated (Piper et al. 2009). The main problem with these techniques is the lack of standardization, so their use in clinical practice is problematic.

Another strategy is based on the use of commercial kits, which must be modified for use in prosthetic joint infections because of the requirement for sonication of the retrieved implant. Commercial tests are designed and standardized for use in daily routines of different laboratories. We used a method similar to that described by Achermann et al. (2010). In our study, a specific commercial kit for commonly isolated pathogens from positive blood cultures was used for identification of bacteria from sonicated implants, based on PCR amplification of a broad-range target followed by hybridization to membranes loaded with different DNA probes. In the kit used by Achermann et al. (2010), some important orthopedic pathogens such as *Propionibacterium sp., Corynebacterium sp. or S. lugdunensis* cannot be detected. Despite this limitation, the modification developed allowed us to increase the sensitivity of the test in the diagnosis of microorganisms from different orthopedic implants. We included prosthetic joint implants as well as devices used in the treatment of fractures; the latter have never been evaluated with molecular techniques previously. We found that detection by molecular analysis increased the number of positive samples found in prosthetic joint implants, as has also been described by others (Tunney et al. 1999, Achermann et al. 2010). We were unable to find any differences between patients who had undergone previous antibiotic treatment and those who had not, in contrast to what has been reported previously (Achermann et al. 2010). This lack of statistical difference between groups was also seen in the numbers of colonies detected from positive samples using a quantitative culture protocol.

We also detected a low number of potential “contaminants”. It has been argued that the use of plastic bags is associated with an unacceptable amount of contamination (Trampuz et al. 2006). In contrast, the number of contaminants in our series appears to have been reasonably low, most of them being isolated from patients who were not diagnosed clinically as being infected, and with low counts, so the actual significance is, at most, doubtful. The protocol we followed included an exchange of the distilled water used for each sonication process, with inspection of the sterile plastic bags before and after sonication. The use of bags allows easy handling of the sample under sterile conditions, particularly in the case of sonication of large implants—which may be difficult with some nails and long stems in rigid plastic containers. Even so, the interpretation of these isolates must be done with caution, because uncommon organisms have been described as being the cause of some implant-related infections, and their isolation cannot be automatically defined as “contamination”.

One aspect of our study that has not been reported previously is the evaluation of the technique for patient diagnosis, rather than for comparison of sample results. More specifically, we were interested in evaluation of a molecular biological technique combined with a sonication protocol for patient diagnosis. In this sense, the number of patients diagnosed using sonication followed by PCR-hybridization increased by one tenth in patients with prosthetic joint infections, compared to the number of patients diagnosed by sonication and conventional culture, and reached 90% of all patients with a diagnosis of prosthetic joint infection. This increase is important: specific antimicrobials can be used, which lead to better management of patients. The molecular kit even permitted detection of some resistance mechanisms, which was not evaluated in this study but which may be useful for therapy.

Molecular detection of pathogens in patients without any clinical diagnosis of infection remains an interpretation challenge. It has been claimed that these cases are, in fact, true implant-related infections that are subclinical, or that are considered aseptic loosening or “painful nails” (Nelson et al. 2005, Marin et al. 2012). In our series, the number of positive results increased in these patients relative to patients who were originally diagnosed as being as clinically infected. Other authors have considered such results to be potential contaminations; they performed modifications to the technique (sonication and molecular biology), which lowered sensitivity and gave results similar to that obtained with conventional methods (Bjerkan et al. 2012). In our opinion, interpretation of these results should be done with caution, especially when common true pathogens such as *S. pyogenes* or *S. aureus* are detected. Reduction of sensitivity is not a good approach, however, because it leads to loss one of the main advantages of these techniques: the increase in sensitivy.

When we analyzed the data obtained from patients with osteosynthesis implants, we obtained different results from those found for prosthetic joints. No clear improvement on diagnosis could be evidenced, probably due to differences in pathogenesis between the 2 groups. The fact that there were many polymicrobial infections in this group and that they included anaerobes in many cases (which are not detected by the kit) would probably explain these findings.

In conclusion, despite the limitations of the kit, its use allowed us to increase the number of patients who were diagnosed as having prosthetic joint infections. Its use appeared to be less valuable in patients with infected osteosynthesis-related implants. Detection of pathogens in patients with no specific signs and symptoms of infection must be interpreted with caution, and these should not be automatically disregarded as contaminants.

## References

[CIT0001] Achermann Y, Vogt M, Leunig M, Wust J, Trampuz A (2010). Improved diagnosis of periprosthetic joint infection by multiplex PCR of sonication fluid from removed implants. J Clin Microbiol.

[CIT0002] Berbari EF, Marculescu C, Sia I, Lahr BD, Hanssen AD, Steckelberg JM (2007). Culture-negative prosthetic joint infection. Clin Infect Dis.

[CIT0003] Bjerkan G, Witso E, Nor A, Viset T, Loseth K, Lydersen S (2012). A comprehensive microbiological evaluation in fifty-four patients undergoing revision surgery due to prosthetic joint loosening. J Med Microbiol.

[CIT0004] Clinical_Laboratory_Standards_Institute_(CLSI) (2009). Performance Standards for Antimicrobial Disk Susceptibility Tests; Approved Standards-Tenth Edition. CLST Document M02-A10.

[CIT0005] Cordero-Ampuero J, Esteban J, Garcia-Cimbrelo E, Munuera L, Escobar R (2007). Low relapse with oral antibiotics and two-stage exchange for late arthroplasty infections in 40 patients after 2-9 years. Acta Orthop.

[CIT0006] Costerton JW (2005). Biofilm theory can guide the treatment of device-related orthopaedic infections. Clin Orthop.

[CIT0007] De Man FHR, Graber P, Lüem M, Zimmerli W, Ochsner PE, Sendi P (2009). Broad-range PCR in selected episodes of prosthetic joint infection. Infection.

[CIT0008] Del Pozo JL, Patel R (2009). Clinical practice. Infection associated with prosthetic joints. N Engl J Med.

[CIT0009] Dempsey KE, Riggio MP, Lennon A, Hannah VE, Ramage G, Allan D (2007). Identification of bacteria on the surface of clinically infected and non-infected prosthetic hip joints removed during revision arthroplasties by 16S rRNA gene sequencing and by microbiological culture. Arthritis Res Ther.

[CIT0010] Dora C, Altwegg M, Gerber C, Bottger EC, Zbinden R (2008). Evaluation of conventional microbiological procedures and molecular genetic techniques for diagnosis of infections in patients with implanted orthopedic devices. J Clin Microbiol.

[CIT0011] Eigner U, Weizenegger M, Fahr AM, Witte W (2005). Evaluation of a rapid direct assay for identification of bacteria and the mec A and van genes from positive-testing blood cultures. J Clin Microbiol.

[CIT0012] Esteban J, Gomez-Barrena E, Cordero J, Martin-de-Hijas NZ, Kinnari TJ, Fernandez-Roblas R (2008). Evaluation of quantitative analysis of cultures from sonicated retrieved orthopedic implants in diagnosis of orthopedic infection. J Clin Microbiol.

[CIT0013] Fihman V, Hannouche D, Bousson V, Bardin T, Liote F, Raskine L (2007). Improved diagnosis specificity in bone and joint infections using molecular techniques. J Infect.

[CIT0014] Gallo J, Kolar M, Dendis M, Loveckova Y, Sauer P, Zapletalova J (2008). Culture and PCR analysis of joint fluid in the diagnosis of prosthetic joint infection. New Microbiol.

[CIT0015] Kobayashi N, Procop GW, Krebs V, Kobayashi H, Bauer TW (2008). Molecular identification of bacteria from aseptically loose implants. Clin Orthop.

[CIT0016] Marin M, Garcia-Lechuz JM, Alonso P, Villanueva M, Alcala L, Gimeno M (2012). The role of universal 16S rRNA gene PCR and sequencing in the diagnosis of prosthetic joint infection. J Clin Microbiol.

[CIT0017] Moojen DJ, Spijkers SN, Schot CS, Nijhof MW, Vogely HC, Fleer A (2007). Identification of orthopaedic infections using broad-range polymerase chain reaction and reverse line blot hybridization. J Bone Joint Surg (Am).

[CIT0018] Nelson CL, McLaren AC, McLaren SG, Johnson JW, Smeltzer MS (2005). Is aseptic loosening truly aseptic?. Clin Orthop.

[CIT0019] Piper KE, Jacobson MJ, Cofield RH, Sperling JW, Sanchez-Sotelo J, Osmon DR (2009). Microbiologic diagnosis of prosthetic shoulder infection by use of implant sonication. J Clin Microbiol.

[CIT0020] Riggio MP, Dempsey KE, Lennon A, Allan D, Ramage G, Bagg J (2010). Molecular detection of transcriptionally active bacteria from failed prosthetic hip joints removed during revision arthroplasty. Eur J Clin Microbiol Infect Dis.

[CIT0021] Sauer P, Gallo J, Kesselova M, Kolar M, Koulakova D (2005). Universal primers for detection of common bacterial pathogens causing prosthetic joint infection. Biomed Pap Med Fac Univ Palacky Olomouc Czech Repub.

[CIT0022] Trampuz A, Osmon DR, Hanssen AD, Steckelberg JM, Patel R (2003). Molecular and antibiofilm approaches to prosthetic joint infection. Clin Orthop.

[CIT0023] Trampuz A, Piper KE, Hanssen AD, Osmon DR, Cockerill FR, Steckelberg JM (2006). Sonication of explanted prosthetic components in bags for diagnosis of prosthetic joint infection is associated with risk of contamination. J Clin Microbiol.

[CIT0024] Trampuz A, Piper KE, Jacobson MJ, Hanssen AD, Unni KK, Osmon DR (2007). Sonication of Removed Hip and Knee Prostheses for Diagnosis of Infection. N Engl J Med.

[CIT0025] Tunney MM, Patrick S, Curran MD, Ramage G, Hanna D, Nixon JR (1999). Detection of prosthetic hip infection at revision arthroplasty by immunofluorescence microscopy and PCR amplification of the bacterial 16S rRNA gene. J Clin Microbiol.

[CIT0026] Vandercam B, Jeumont S, Cornu O, Yombi JC, Lecouvet F, Lefevre P (2008). Amplification-based DNA analysis in the diagnosis of prosthetic joint infection. J Mol Diagn.

